# Supplementary material for: Why are viral genomes so fragile? The bottleneck hypothesis
Source: PLoS Comput Biol. 2021 Jul 8;17(7):e1009128. doi: 10.1371/journal.pcbi.1009128 (PMC8291636; doi:10.1371/journal.pcbi.1009128)
Supplement: S1 Text — (PDF) [file pcbi.1009128.s001.pdf]

Supplementary Information for

Why are viral genomes so fragile? The bottleneck hypothesis

Nono S. C. Merleau, Sophie Pénisson, Philip J. Gerrish, Santiago F. Elena, Matteo Smerlak,

A Muller's ratchet in expanding populations

Branching process setting

The composition of the viral population is determined by the number of virions carrying 0, 1, 2, *etc.* deleterious mutations. Its evolution is modeled by a branching process [1]: each viral particle in generation n randomly produces particles in generation $n+1$, independently of the rest of the population and according to some probability distribution which depends solely on the "type" of the particle. Since there are no back mutations, the resulting process $(\mathbf{X}_n)_{n\in\mathbb{N}}$ is a reducible branching process with infinitely many types, where $\mathbf{X}_n = (X_{n,i})_{i\in\mathbb{N}}$ is the population composition at time n, and $X_{n,i}$ the number of i-mutants (particles carrying i mutations) at this time. All the properties of the branching process are contained in its offspring generating functions $F_0, F_1, \ldots$. The generating function F_i determines the distribution of the number of various mutants to be produced by an i-mutant. By definition, for $\mathbf{r} \in [0,1]^{\mathbb{N}}$, $F_i(\mathbf{r})$ is the expected value of $r_0^{X_{1,0}} r_1^{X_{1,1}} \ldots$ when the process is initiated by one i-mutant. Because there are no back mutations, it solely depends on r_{i+j}, $j \geqslant 0$, and we write $F_i(r_i, r_{i+1}, \ldots)$.

We assume throughout this paper that the average number of $(i+j)$-mutants produced by an i-mutant is $w_i e^{-u} u^j / j!$. This is achieved for instance if the total number of offspring of an i-mutant follows a Poisson distribution $\mathcal{P}(w_i)$, and if each of its offspring accumulates a number of additional deleterious mutations according to $\mathcal{P}(u)$. In this particular case, the i-th generating function is

$$F_i(r_i, r_{i+1}, \ldots) = \sum_{k\in\mathbb{N}} e^{-w_i} \frac{w_i^k}{k!} \sum_{\substack{k_i, k_{i+1}, \ldots \in \mathbb{N} \\ \sum_{j\geqslant 0} k_{i+j} = k}} \frac{k!}{k_i! k_{i+1}! \ldots} \prod_{j\geqslant 0} \left(e^{-u} \frac{u^j}{j!} r_{i+j} \right)^{k_{i+j}}$$

which simplifies to

$$F_i(r_i, r_{i+1}, \ldots) = \exp\left[-w_i \left(1 - e^{-u} \sum_{j\geqslant 0} \frac{u^j}{j!} r_{i+j} \right) \right]. \tag{1}$$

Survival probability

Let $m_i = w_i e^{-u}$ the average number of i-mutants produced by an i-mutant. It follows from a general result of branching processes theory that if $m_i \leqslant 1$, any population starting with one i-mutant is doomed to extinction; if $m_i > 1$, this population has positive survival probability. Let $K = \max\{k : m_k > 1\}$ the largest number of mutations which can be carried by an individual without leading to its progeny's

extinction, and call *supercritical* any k-mutant with $k \leqslant K$. By independence of the lineages, the fate of the population $(\mathbf{X}_n)_{n\in\mathbb{N}}$ only depends on the initial numbers of supercritical mutants $\mathbf{n} = (X_{0,0}, \ldots, X_{0,K})$. First, we compute for each $k \leqslant K$ the *partial* extinction probability $p_{\text{ext},k}(\mathbf{n})$ of all mutants up to type k, given the initial condition $\mathbf{n}$, namely the probability of $\{X_{n,i} \to 0,\ \forall i \leqslant k\}$. It corresponds to the extinction probability of the reducible branching process with finite set of types $\{0, 1, \ldots, k\}$ and generating functions $F_i(r_i, \ldots, r_k, 1, 1, \ldots)$, $i \leqslant k$. As such [2], the partial extinction probability is given by

$$p_{\text{ext},k}(\mathbf{n}) = \prod_{i=0}^{k} q_{ki}^{n_i}, \tag{2}$$

where $(q_{k0}, \ldots, q_{kk})$ is the smallest nonnegative solution of the system of equations

$$F_i(q_{ki}, \ldots, q_{kk}, 1, 1, \ldots) = q_{ki}, \quad 0 \leqslant i \leqslant k, \tag{3}$$

(see Eq (19) for an explicit solution when F_i is given by Eq (1)). The overall survival and extinction probabilities of the population are then obtained as

$$p_{\text{surv}}(\mathbf{n}) = 1 - p_{\text{ext}}(\mathbf{n}) = 1 - p_{\text{ext},K}(\mathbf{n}). \tag{4}$$

Muller's ratchet click probabilities

For a population starting with supercritical mutants $\mathbf{n}$ and $k \leqslant K$, let $p_k(\mathbf{n})$ the probability that the fittest surviving individuals carry k mutations. Equivalently, it is the probability that Muller's ratchet clicks $k - k_0$ times, where $k_0 = \min\{i :\ n_i > 0\}$ is the fittest initial supercritical type. It corresponds to event $\{X_{n,k} \nrightarrow 0,\ X_{n,i} \to 0,\ \forall i < k\}$ (all i-mutants with $i < k$ go extinct but k-mutants survive), therefore

$$\begin{cases} p_0(\mathbf{n}) = 1 - p_{\text{ext},0}(\mathbf{n}), \\ p_k(\mathbf{n}) = p_{\text{ext},k-1}(\mathbf{n}) - p_{\text{ext},k}(\mathbf{n}), \end{cases} \tag{5}$$

It comes in particular $p_{\text{surv}}(\mathbf{n}) = \sum_{k=0}^{K} p_k(\mathbf{n})$, consistent with the fact that any click beyond the threshold K leads to extinction.

Mutant spectra and asymptotic mean fitness

The late-time composition of the population is completely determined by the number of clicks of the ratchet. On the event Ω_k that the type of its fittest surviving individuals is k, the asymptotic behavior of the population can be described with the help of the branching process $\mathbf{Z}_n$ with offspring generating functions $G_i = F_{i+k}$, $i \geqslant 0$, which only considers particles with type greater than k. To study this process we use results from the theory on decomposable branching processes [3], also used in [4] in the context of a viral population. The offspring mean matrix of the process is the upper triangular matrix $\mathbf{M} = (m_{k+i,k+j})_{i,j\geqslant 0}$, where m_{ij} is the average number of j-mutants born from an i-mutant in the original process $\mathbf{X}_n$. Its largest eigenvalue is $m_k > 1$, and $\mathbf{u} = (1, 0, \ldots)$, $\mathbf{v} = \left((u/s_d)^i/i!\right)_{i\geqslant 0}$ are respectively non-negative right and left eigenvectors of $\mathbf{M}$ corresponding to m_k, satisfying $\mathbf{u} \cdot \mathbf{v}' = 1$. Assuming $\mathbf{Z}_0 = (1, 0, \ldots)$, then $m_k^{-n}\mathbf{Z}_n$ almost surely converges to $W\mathbf{v}$, where W is a non-negative random variable with mean value 1, and $\{W = 0\} = \{Z_{n,0} \to 0\}$. If $\mathbf{Z}_0 = (z_0, 0, \ldots)$, then by independence of the lineages $m_k^{-n}\mathbf{Z}_n \to W^{*z_0}\mathbf{v}$, where W^{*z_0} is the sum of z_0 independent copies of W. The limit remains the same if $\mathbf{Z}_0 = (z_0, z_1, \ldots)$, since the z_i i-lineages with $i > 0$ have growth rate $m_{k+i} < m_k$ and tend to 0 if normalized by m_k^n.

It follows that for any initial value $\mathbf{Z}_0$, $\mathbf{Z}_n/\sum_j Z_{n,j} \to e^{-u/s_d}\mathbf{v}$ almost surely on the event $\{Z_{n,0} \nrightarrow 0\}$.

Let $N_n = \sum_i X_{n,i}$ be the total population size at time n and let $f_i = e^{-u/s_d}(u/s_d)^i/i!$. We deduce from what precedes that the fraction of i-mutants $(i \geqslant k)$ in the population satisfies

$$\lim_n \frac{X_{n,i}}{N_n} = f_{i-k} \quad \text{almost surely on } \Omega_k, \tag{6}$$

while this proportion converges to 0 if $i < k$. Since $(\Omega_k)_{0 \leqslant k \leqslant K}$ forms a partition of the survival set, the previous result can be stated as follows (setting the frequency to 0 if $N_n = 0$):

$$\lim_n \frac{X_{n,i}}{N_n} = Y_i \quad \text{almost surely}, \tag{7}$$

where Y_i is a random variable equal to f_{i-k} with probability $p_k(\mathbf{n})$, $k \leqslant \min(i, K)$, and equal to 0 with probability $p_{\text{ext}}(\mathbf{n}) + \sum_{k=i+1}^{K} p_k(\mathbf{n})$. Therefore, the mean population fitness satisfies

$$\lim_n \sum_{i \geqslant 0} w_i \frac{X_{n,i}}{N_n} = \overline{w}_\infty \quad \text{almost surely}, \tag{8}$$

where $\overline{w}_\infty = \sum_{i \geqslant 0} w_i Y_i$ is a random variable equal to $w_k e^{-u}$ with probability $p_k(\mathbf{n})$, $0 \leqslant k \leqslant K$, and equal to 0 with probability $p_{\text{ext}}(\mathbf{n})$. It follows that the expected asymptotic population mean fitness is given by

$$\begin{cases} \mathbb{E}(\overline{w}_\infty) = e^{-u} \sum_{k=0}^{K} w_k p_k(\mathbf{n}), \\ \mathbb{E}(\overline{w}_\infty \mid \text{survival}) = e^{-u} \sum_{k=0}^{K} w_k p_k(\mathbf{n}) / p_{\text{surv}}(\mathbf{n}). \end{cases} \tag{9}$$

B Survival through multiple bottlenecks

Markov chain model

In order to assess the effect of successive bottleneck events on the viral population, we introduce a stochastic model describing the evolution of the random composition of the population after each size reduction event. If the population reaches its carrying capacity C, a sample of size B is taken, resulting in a new founding population. As described earlier, the fate of this new population only depends on its initial numbers of supercritical mutants $\mathbf{n}$, with $|\mathbf{n}| = \sum_{k=0}^{K} n_k \leqslant B$ (the sample might include some particles which are not supercritical). We choose C large enough such that (i) the probability of not reaching C is close to the extinction probability of the population, (ii) if it reaches C, the mutant frequencies in the population follow the asymptotic random distribution Eq (7). The evolution of the viral population going through these multiple size reductions is then entirely determined by the successive states $\mathbf{n}_0, \mathbf{n}_1, \ldots$, where $\mathbf{n}_b$ describes the supercritical fitness classes after b bottleneck events. We describe this random evolution via a Markov chain with state space $\mathcal{S} = \left\{\mathbf{n} \in \mathbb{N}^{K+1},\ |\mathbf{n}| \leqslant B\right\}$, and transition probability from state $\mathbf{m}$ to state $\mathbf{n}$:

$$P(\mathbf{m} \to \mathbf{n}) = \sum_{k=0}^{K} Q_k(\mathbf{n})\, p_k(\mathbf{m}) + \mathbf{1}_{\mathbf{n}=\mathbf{0}} p_{ext}(\mathbf{m}), \tag{10}$$

where $Q_k(\mathbf{n})$ is the probability of obtaining $\mathbf{n}$ supercritical mutants in the sample of size B, if the fittest surviving individuals in the population founded by $\mathbf{m}$ are of type k (event of probability $p_k(\mathbf{m})$). It follows from Eq (6) that on the latter event, the asymptotic proportion of non-supercritical mutants is $1 - \sum_{i=0}^{K-k} f_i$, while the

asymptotic proportion of supercritical i-mutants is f_{i-k} if $i \geqslant k$, and 0 otherwise. The random composition of the sample of size B thus follows a multinomial distribution with B trials and success probabilities $\{0, \ldots, 0, f_0, \ldots, f_{K-k}, 1 - \sum_{i=0}^{K-k} f_i\}$, the last coordinate corresponding to the non-supercritical mutants. Therefore, the probability of getting $\mathbf{n}$ supercritical mutants is

$$Q_k(\mathbf{n}) = \frac{B! 0^{\sum_{i=0}^{k-1} n_i} \left(1 - \sum_{i=0}^{K-k} f_i\right)^{B-|\mathbf{n}|} \prod_{i=0}^{K-k} f_i^{n_{i+k}}}{n_0! \ldots n_K! \left(B - |\mathbf{n}|\right)!}. \tag{11}$$

Extinction probability under multiple bottlenecks

Let $\mathbf{P} = \left(P\left(\mathbf{m} \to \mathbf{n}\right)\right)_{\mathbf{m},\mathbf{n} \in \mathcal{S}}$ the stochastic matrix of this Markov chain, and $\mathbf{P}^b$ its b-th power. Then the probability for a viral population with initial state $\mathbf{n}_0$ to become extinct after going through at most b bottlenecks is

$$p_{\text{bottlenecks},b}\left(\mathbf{n}_0\right) = \mathbf{P}^b\left(\mathbf{n}_0, \mathbf{0}\right), \tag{12}$$

i.e. entry $(\mathbf{n}_0, \mathbf{0})$ of matrix $\mathbf{P}^b$. This probability can thus be computed explicitly as soon as the extinction and click probabilities p_{ext} and p_k are known, which is the case if for instance the numbers of offspring and of accumulated mutations follow Poisson distributions (see Eq (19)).

C Epidemiology of genetic fragility

Incubation period distribution

How long does it take for the in-host population carried by an exposed individual E to reach size C? Assuming C is large enough, this random time can be approximated thanks to the asymptotic behavior of the population. Let $\mathbf{n}$ the initial numbers of supercritical viral particles in the in-host population. As described earlier, if the population does not go extinct, its limit behavior depends on the type k of its fittest surviving particles. The distribution of the incubation period thus depends on $\mathbf{n}$ and k. When a host is infected, we therefore specify these parameters $\mathbf{n}$ and k and denote by $\tau_{\mathbf{n},k}$, $E_{\mathbf{n},k}$ and $I_{\mathbf{n},k}$ the corresponding incubation period, exposed and infectious states.

More specifically, when a susceptible S meets an infectious individual $I_{\mathbf{m},l}$, it receives $\mathbf{n}$ supercritical viral particles with probability $Q_l(\mathbf{n})$, $\mathbf{n} \in \mathcal{S}$, given by Eq (11). The susceptible individual then either remains in state S with probability $p_{\text{ext}}(\mathbf{n})$, or enters in exposed state $E_{\mathbf{n},k}$ with probability $p_k(\mathbf{n})$, $0 \leqslant k \leqslant K$, given by Eq (5). If so, it remains in $E_{\mathbf{n},k}$ for $\tau_{\mathbf{n},k}$ time steps Eq (13) before entering infectious state $I_{\mathbf{n},k}$. Let N_n the total viral population size carried by $E_{\mathbf{n},k}$ at time n. We deduce from previously mentioned results that $m_k^{-n} N_n$ converges almost surely to $e^{u/s_d} W_{\mathbf{n},k}$, where $W_{\mathbf{n},k}$ is a positive random variable whose distribution is given by Eq (15), and explicitly by Eq (21) in the particular case of Poisson distributed numbers of offspring and mutations described by Eq (1). For C large enough, the time needed for N_n to reach size C can consequently be approximated by

$$\tau_{\mathbf{n},k} = \left\lceil \frac{\ln\left(C/W_{\mathbf{n},k}\right) - u/s_d}{\ln m_k} \right\rceil. \tag{13}$$

In the general setting, the random variable $W_{\mathbf{n},k}$ is approximately the sum of L independent copies of V, where (i) $L \geqslant 1$ is the random number of surviving k-lineages (i.e. a line of descent of a k-mutant with no k-mutants as ancestors), and (ii) V is the limiting distribution of $m_k^{-n} X_n$ on its survival set, if $(X_n)_n$ is a monotype branching process modeling only k-mutants. Although not all explicit, we can state the following general results on L and V.

(i) Let k_0 the fittest initial type in $\mathbf{n}$. If Muller's ratchet does not click, i.e. if $k = k_0$, then the number L of surviving k-lineages is at least 1 and at most n_k. Since the survival probability of a k-lineage is $1 - q_{kk}$, L follows a binomial distribution with parameters n_k and $1 - q_{kk}$, conditioned on being positive. If $k > k_0$ however, there might be more than n_k surviving k-lineages (namely also those stemming from the $\sum_{i=k_0}^{k-1} n_i$ initial mutants). We therefore approximate the maximum number of surviving k-lineages by the average number of k-mutants born from $\mathbf{n}$ after one time-step, conditioned on the event that the surviving individuals are of type k (which we denote Ω_k). This average is equal to $\sum_{i=k_0}^{k} n_i \tilde{m}_{ik}$, where $\tilde{m}_{ik}$ is the mean number of k-mutants born from one i-mutant, conditionally on Ω_k. In the following computation, the subscript i indicates that the process starts with one i-mutant, and $\mathbf{q}_k$ stands for the infinite vector $(q_{k0}, \ldots, q_{kk}, 1, \ldots)$ defined in Eq (4). It comes from Eq (2) and Eq (5) that $\mathbb{P}_i(\Omega_k) = q_{k-1,i} - q_{ki}$, and that for any infinite vector $\mathbf{x}$, using the notation $\mathbf{x}^{\mathbf{y}} = \prod_{i\in\mathbb{N}} x_i^{y_i}$, $\mathbb{P}_{\mathbf{x}}(\Omega_k) = \mathbf{q}_{k-1}^{\mathbf{x}} - \mathbf{q}_k^{\mathbf{x}}$. With this notation, $F_i(\mathbf{r}) = \mathbb{E}_i\left(\mathbf{r}^{\mathbf{X}_1}\right)$ and $r_k \frac{\partial F_i}{\partial r_k}(\mathbf{r}) = \mathbb{E}_i\left(X_{1k}\mathbf{r}^{\mathbf{X}_1}\right)$. As a result, the mean $\tilde{m}_{ik}$ is obtained as

$$\begin{aligned}\tilde{m}_{ik} = \mathbb{E}_i\left(X_{1k} \mid \Omega_k\right) &= \frac{\mathbb{E}_i\left(X_{1k}\mathbb{P}_{\mathbf{X}_1}(\Omega_k)\right)}{\mathbb{P}_i(\Omega_k)} \\ &= \frac{\mathbb{E}_i\left(X_{1k}\mathbf{q}_{k-1}^{\mathbf{X}_1}\right) - \mathbb{E}_i\left(X_{1k}\mathbf{q}_k^{\mathbf{X}_1}\right)}{q_{k-1,i} - q_{ki}} \\ &= \frac{\frac{\partial F_i}{\partial r_k}(\mathbf{q}_{k-1}) - q_{kk}\frac{\partial F_i}{\partial r_k}(\mathbf{q}_k)}{q_{k-1,i} - q_{ki}}.\end{aligned} \tag{14}$$

(ii) Let $(X_n)_n$ a branching process with $X_0 = 1$ and generating function $F(r) := F_k(r, 1, 1, \ldots)$, $r \in [0, 1]$. It is therefore supercritical with mean offspring number $m_k > 1$. From the theory on monotype branching processes [1] we know that its extinction probability is the smallest nonnegative solution of $F(r) = r$, which is by definition q_{kk} given by Eq (4). Moreover, $m_k^{-n} X_n \to W$ almost surely, where W is a nonnegative random variable whose Laplace transform $\varphi(s) = \mathbb{E}\left(e^{-sW}\right)$ is the unique solution of $\varphi(m_k s) = F(\varphi(s))$. Then the desired random variable V is distributed as W conditioned on $W > 0$. Since $\mathbb{P}(W = 0) = q_{kk}$, the Laplace transform of V is $\phi(s) = (\varphi(s) - q_{kk}) / (1 - q_{kk})$.

To summarize, we assume that the random variable $W_{\mathbf{n},k}$ appearing in Eq (13) is given by

$$W_{\mathbf{n},k} = \sum_{i=1}^{L} V^{(i)}, \tag{15}$$

where L is a random integer such that

$$\mathbb{P}(L = l) = \binom{\tilde{n}_k}{l} \frac{(1 - q_{kk})^l \, q_{kk}^{\tilde{n}_k - l}}{1 - q_{kk}^{\tilde{n}_k}}, \quad 1 \leqslant l \leqslant \tilde{n}_k, \tag{16}$$

with

$$\tilde{n}_k = \begin{cases} n_k, & k = k_0, \\ \left\lceil \sum_{i=k_0}^{k} n_i \tilde{m}_{ik} \right\rceil, & k > k_0, \end{cases} \tag{17}$$

and where the $V^{(i)}$ are independent copies of the random variable V with Laplace transform

$$\phi(s) = \frac{\varphi(s) - q_{kk}}{1 - q_{kk}}, \tag{18}$$

φ being the unique Laplace transform solution of $\varphi\left(m_k s\right) = F_k\left(\varphi\left(s\right), 1, 1, \ldots\right)$

D Explicit formulas

Poisson distributed number of offspring

In the particular case of a Poisson distributed number of offspring and mutations (with generating functions Eq (1)), an exact computation of $\left(q_{k0}, \ldots, q_{kk}\right)$ can be found, leading to explicit expressions of the extinction and Muller's ratchet click probabilities Eq (4)-Eq (5) and of the expected asymptotic population mean fitness Eq (9). Indeed, system Eq (3) becomes

$$\exp\left[-m_i\left(\sum_{j=i}^{k}\frac{u^{j-i}}{(j-i)!}\left(1-q_{kj}\right)\right)\right] = q_{ki}, \quad 0 \leqslant i \leqslant k,$$

leading to

$$\begin{cases} q_{kk} = -\dfrac{1}{m_k} W\left[-m_k e^{-m_k}\right], \\ q_{ki} = -\dfrac{1}{m_i} W\left[-m_i e^{-m_i\left(1+\sum_{j=i+1}^{k} u^{j-i}\left(1-q_{kj}\right)/(j-i)!\right)}\right], \quad 0 \leqslant i \leqslant k-1, \end{cases} \tag{19}$$

where $W\left[\cdot\right]$ stands for the principal solution of the Lambert W function.

In this particular case, we can also compute Eq (14) and Eq (18), and therefore provide the explicit distribution Eq (15) of $W_{\mathbf{n},k}$ involved in the incubation period of the exposed state Eq (13). Indeed, with F_i given by Eq (1), we have $\frac{\partial F_i}{\partial r_k}\left(\mathbf{r}\right) = m_{ik} F_i\left(\mathbf{r}\right)$, where $m_{ik} = w_i e^{-u} u^{k-i} / \left(k-i\right)!$. In addition, since $\mathbf{q}_k$ satisfies by definition $F_i\left(\mathbf{q}_k\right) = q_{ki}$, Eq (14) becomes

$$\tilde{m}_{ik} = m_{ik}\frac{q_{k-1,i} - q_{kk}q_{ki}}{q_{k-1,i} - q_{ki}}. \tag{20}$$

Finally, we show that, approximately, the random variable V involved in Eq (15) follows an exponential distribution with parameter $1 - q_{kk}$. Indeed, the latter means that $\phi\left(s\right) = \frac{1-q_{kk}}{1-q_{kk}+s}$, and thus $\varphi\left(s\right) = 1 - \frac{\left(1-q_{kk}\right)s}{1-q_{kk}+s}$. We have on the one hand

$$\varphi\left(m_k s\right) = 1 - m_k\frac{\left(1-q_{kk}\right)s}{1-q_{kk}+s},$$

while on the other hand

$$F_k\left(\varphi\left(s\right), 1, \ldots\right) = \exp\left[-m_k\frac{\left(1-q_{kk}\right)s}{1-q_{kk}+s}\right],$$

hence for m_k close to 1, $\varphi\left(m_k s\right) \approx F_k\left(\varphi\left(s\right), 1, \ldots\right)$, leading to our rough approximation. As the sum of independent exponentially distributed variables, $W_{\mathbf{n},k}$ thus follows a Gamma distribution with shape and rate parameters

$$W_{\mathbf{n},k} \sim \mathrm{Gamma}\left(L, 1-q_{kk}\right) \tag{21}$$

where L is given by Eq (16) with

$$\tilde{n}_k = \begin{cases} n_k, & k = k_0, \\ \left\lceil \displaystyle\sum_{i=k_0}^{k} n_i m_{ik}\frac{q_{k-1,i} - q_{kk}q_{ki}}{q_{k-1,i} - q_{ki}} \right\rceil, & k > k_0. \end{cases} \tag{22}$$

Binary reproduction

Other reproduction mechanisms can be considered, with the same key assumption that the average number of $(i+j)$-mutants produced by an i-mutant is $w_i e^{-u} u^j / j!$. For example, we can assume a binary reproduction (although less relevant for viral populations) where an i-mutant either dies, or produces two offspring with probability $w_i/2$, each of them accumulating a random number of additional mutations following $\mathcal{P}(u)$. In this case, the generating function is quadratic

$$F_i\left(r_i, r_{i+1}, \ldots\right) = 1 - \frac{w_i}{2} + \frac{w_i}{2}\left(\sum_{j\in\mathbb{N}} e^{-u}\frac{u^j}{j!} r_{i+j}\right)^2,$$

and system Eq (3) comes down to

$$1 - \frac{w_i}{2} + \frac{w_i}{2}\left(1 - \sum_{j=i}^{k} e^{-u}\frac{u^{j-i}}{(j-i)!}\left(1-q_{kj}\right)\right)^2 = q_{ki}, \quad 0 \leqslant i \leqslant k,$$

which can be solved explicitly as well.

References

1. Athreya KB, Ney PE. Branching processes. Dover Publications, Inc., Mineola, NY; 2004.

2. Hautphenne S. Extinction probabilities of supercritical decomposable branching processes. J Appl Probab. 2012;49(3):639–651. doi:10.1239/jap/1346955323.

3. Kesten H, Stigum BP. Limit theorems for decomposable multi-dimensional Galton-Watson processes. Journal of Mathematical Analysis and Applications. 1967;17(2):309 – 338. doi:https://doi.org/10.1016/0022-247X(67)90155-2.

4. Antoneli F, Bosco F, Castro D, MARIO JANINI L. Viral evolution and adaptation as a multivariate branching process. In: BIOMAT 2012. World Scientific; 2013. p. 217–243. Available from: https://www.worldscientific.com/doi/abs/10.1142/9789814520829_0013.
