# Supplementary material for: Why are viral genomes so fragile? The bottleneck hypothesis
Source: PLoS Comput Biol. 2021 Jul 8;17(7):e1009128. doi: 10.1371/journal.pcbi.1009128 (PMC8291636; doi:10.1371/journal.pcbi.1009128)
Supplement: Attachment — Submitted filename: Why_are_viral_genomes_so_fragile - response.pdf [file pcbi.1009128.s002.pdf]

Response to the reviewers

We thank the reviewers for their critical assessment of our work. In the following we address their concerns point by point.

Reviewer 1

Reviewer Point P 1.1 — Figure 1, and the model specification (only deleterious mutations, high mutation rate, no back mutations) seem to suggest that fragile viruses will be selected for in the long run. At least, so long as $w_0 e^{-u} > 1$. Bottlenecks only act to speed up the process. Is there a parameter space where bottlenecks are required for fragility to be advantageous? Figure 4 hints at this idea and shows for B=5, w0-1.2, and u=0.05 The survival probability after 1 bottleneck is fairly flat. Is there a parameter space where fragility is selected against, such that some mechanism of mutational robustness is required?

Reply: Without bottlenecks, robustness is always advantageous. To clarify this point we have added an inset within Fig. 4 showing the dependence of the extinction probability on s_d in the absence of bottlenecks.

Reviewer Point P 1.2 — A range of parameter values is used throughout the figures. The manuscript would be strengthened if the parameter values were discussed and could be connected to those observed in viral populations. It appears that relationships between the parameters are more important than the actual values. For example, survival probability depends on whether or not $we^{-u} > 1$, but this could still be put into biological context.

Reply: Indeed, this was missing. We have added a new paragraph in the Methods section justifying the selection of parameter values based on experimental evidence.

Reviewer Point P 1.3 — Related to the above point, the SEIR modeling shows how under certain epidemiological conditions fragility can out-compete robustness. However, it seems unrealistic for this selection to take place over 1 epidemic, and this impracticality detracts from an otherwise clear example of the "composite fitness" mentioned in the discussion. Some discussion comparing the parameters in these simulations to those seen in real pathogens would either strengthen the argument that viruses are under the same selective pressures as seen in this model or help to emphasize the purpose of the simulations.

Reply: We agree with the reviewer that selection over a single epidemic is over-simplifying a very complex process. We feel however that the paper as it stands can be viewed as a proof of concept, and leave it for future work to investigate more realistic settings. The limitations of our approach is the subject of an extended discussion in the last paragraphs of the paper. Regarding parameters, see our response above.

Reviewer Point P 1.4 — Figure 1 and throughout the text, the fragile individuals are described as having a smaller starting fitness. It seems the starting fitness of both wild types should be the same (w0).

Reply: The starting fitness of both WT is indeed the same throughout the article. In Fig. 1, what differs is the *population mean fitness*, which is w_0 minus the mutational load. That load is larger in fragile populations, and this is why the blue curve (fragile) is initially below the orange curve (robust). To clarify this we changed the label on the y-axis of Fig. 1 to "Population mean fitness".

Reviewer Point P 1.5 — More time could be spent discussing how fragility evolves. It's not clear that bottlenecks need to be the only or even the main force driving fragility. Shorter genomes with overlapping ORFs likely replicate faster and are fragile. Perhaps, multiple selective forces are pushing in the same direction.

Reply: This is true. In the introduction we wrote "Viral RNA genomes generally contain overlapping reading frames, encode for polyproteins that need to be precisely processed post-translationally, and express multifunctional proteins involved in different processes along the infection cycle; these structural properties predict large deleterious effects of most mutations (5, 6), as is indeed observed (30)." This clearly indicates that other effects are potentially at play. To avoid any misunderstanding, we rewrote our result statement as "In this paper we show that the population bottlenecks experienced by RNA viruses contribute to promoting fragile genetic architectures"; the abstract was also edited accordingly. Finally, in response to a related comment from Reviewer 2, we have added a new paragraph in the Discussion dealing with the evolution of alternative sources of mutational robustness in viral populations as well as their dependency on environmental stresses (*i.e.*, the plastogenetic congruence hypothesis).

Reviewer Point P 1.6 — Figure 5) What do the red lines signify?

Reply: It highlights the region of parameter (β, u) where both fragile and robust strains have equal attack rate (i.e $EIR_f/EIR_r = 1$). We have added a sentence in the caption to clarify this.

Reviewer Point P 1.7 — Methods: After Eq 1 the subscripts in the the series $(0, 0, 0, f_0, f_1, ...)$ are unclear. Should it read $(0, 0, 0, f_k, f_{k+1})$, or does i represent the number of mutations more than k?

Reply: To be less confusing, this sentence has been rewritten as "On the event that the fittest surviving individuals carry k mutations, the asymptotic proportion of $k+i$-mutants in the population is $e^{-u/sd}(u/sd)^i/i!$".

Reviewer Point P 1.8 — The SEIR is a nice demonstration of how fragility could be advantageous. It would be a neat (but admittedly unneeded addition) if a parameter space that gave periodic epidemics was used in which this competition could take place over a longer period.

Reply: This is an interesting suggestion. Since we do not know how to find this parameter space at present, we ask for permission to postpone this discussion to a follow-up study.

Reviewer 2

Reviewer Point P 2.1 — I find some similarities between this paper (specifically, the in host evolution) and the analysis of the paper of Stern et al (Stern, A., Bianco, S., Yeh, M. Te, Wright, C.,

Butcher, K., Tang, C., … Andino, R. (2014). Costs and Benefits of Mutational Robustness in RNA Viruses. Cell Reports, 8(4), 1–11. https://doi.org/10.1016/j.celrep.2014.07.011). An important conclusion of that paper, which is limited to the short term dynamic within a few bottleneck events, is that population fragility gives transient advantage in very selective environments. I wonder whether the author do see this effect in their simulations. I would like the authors to comment on similarities and differences between the two studies.

Reply: We thank Dr. Bianco for pointing us to his work published in *Cell Reports* on the effect of differences in host susceptibility (*i.e.*, how harsh or permissive an environment would be) on poliovirus robustness and evolvability. We have added a new full paragraph in the Discussion with two aims: (1) present popular theories for the evolution of robustness/fragility, highlighting the importance of plastogenetic congruence as the most compelling explanation for RNA viruses, and (2) relating our study with his modeling approach, mentioning similarities, and more importantly, differences.

Reviewer Point P 2.2 — I appreciate the multi-type branching process used to generate the populations. However, it is unclear to me whether or not bottlenecks may create environmental "discontinuity", whereas a population may be subject to stronger or weaker pressure (in the way defined by the authors) following a bottleneck event. I would ask the authors to clarify this point and add it (without to much detail, if they like) to the main manuscript. I apologize if I have not found it.

Following my previous comment, since the authors do not define robustness and fragility as property of the environment, I find the epidemiological dynamic unclear. Specifically, if a population transmit from one host to the next, the susceptible host receives n viral particles. My reading of the supplementary information is that there is no additional environmental constraint on the evolving population. However, this host homogeneity is far from being realistic. I would like the authors to comment on this in the manuscript. Given that we have not specifically introduced environmental heterogeneity (in the form of differences in host susceptibility), we can not make a direct comparison between our modeling approach and the one presented in his *Cell Reports* paper.

Reply: Dr. Bianco is absolutely right. Here we are defining robustness/fragility as an inherent property of the viral population and not as a environment-dependent one. This assumption is in line with previous studies by other authors (*e.g.*, Wilke 2001; Krakauer and Plotkin 2002; not to mention the seminal work by Eigen showing the co-called "quasispecies effect") that sought to explore the evolution of mutational robustness in a constant environment. Furthermore, We agree with Dr. Bianco that the assumption of host homogeneity is far from realistic in real viral populations. However, this assumption is also commonly taken as baseline by most epidemiological models. Of course, models can be expanded to incorporate differences in host susceptibility parameters (SEIR with multiple S and E classes). We could hypothesize that host's variability in susceptibility will play two roles in the evolution of virus' fragility: for highly homogeneous host populations (such as for instance plant crops or livestock) fragile viruses will benefit from the advantages described in our study; by contrast, in highly polymorphic host populations, this advantage may vanish in favor of a robust strategy, as suggested by Dr. Bianco's work. This is such an interesting question that we are already exploring it within our modeling framework. Whatever the results, they will be presented in a follow up manuscript.
